# Knowledge, attitude and practices on jigger infestation among household members aged 18 to 60 years: case study of a rural location in Kenya

**Published:** 2012-12-25

**Authors:** Bernard Kimani, Josephat Nyagero, Lawrence Ikamari

**Affiliations:** 1 African Medical and Research Foundation (AMREF), Nairobi, Kenya; 2 Public Health Officer Ministry of Public Health and Sanitation, Nairobi, Kenya; 3 Population Studies and Research Institute, University of Nairobi, Nairobi, Kenya

**Keywords:** Jigger infestation, knowledge attitude and practices, household

## Abstract

**Introduction:**

Jigger infestation is an important but neglected public health problem. The study assessed the knowledge, attitude and practices of household members on jigger infestation, practices and control within Murang'a district, a rural location in Kenya.

**Methods:**

A cross-sectional descriptive study design was used. Structured interview schedules and observation checklist were used to collect quantitative data. A sample size of 271 household members was interviewed. Descriptive and inferential statistics were analyzed and odds ratios computed at 95% confidence interval to determine variables association.

**Results:**

On knowledge, 70.1% acknowledged poor hygiene and sanitation contributes to jigger infestation while 16.6% identified jigger flea as the cause of jigger infestation. Over half (53.9%) reported jiggers are transmissible from person to person. Majority (94.8%) identified signs and symptoms of jigger infestation. Over a quarter (23.6%) reported an infested household member and 18.8% infested persons were confirmed during the study. Many (59.8%) held the opinion that, jigger infested persons are lazy, 26.2% reported they are poor and 12% reported they either have specific blood or are from certain families. Below half (48.7%) believed in myths and misconceptions on jiggers. Majority (90.8%) reported needles/pins were the mostly used jigger removal items followed by thorns 38.7%. About two thirds (62.0%) were not aware of communal jigger prevention and control activities. The Chi-square results showed that, the village, type of house floor and compound maintenance were significantly associated with jigger infestation (p<0.05).

**Conclusion:**

Knowledge on jigger infestation is high but this has not translated to jigger prevention and control in the area.

## Introduction

Jigger flea, also known as sand flea, *Chigoe* or *Tunga penetrans* is an ecto-parasite which causes Tungiasis parasitic condition of humans and animals. The flea affects many impoverished populations living in sub-Saharan Africa, the Caribbean and South America. Hundreds of millions of people are at risk of infection in more than 70 nations, mostly in developing countries. The importance of Tunga infestation is localization in the foot causing serious difficulty in walking, reducing the infected person's ability to work normally. In endemic areas, prevalence ranges from 15-40% [1].

Jigger transmission from one person to another is not possible but it occurs through the insanitary environment. The jigger flea causes debility in resource-poor communities of developing countries. The flea survives best in sandy and dusty environments. Poverty and powerlessness or inability to do anything about it is the greatest cause of ill health among communities. Tungiasis occurs in resource poor countries in the Caribbean, South America and Africa. In some communities, the prevalence may be as high as 50% in the general population [2]. Tungiasis is usually considered an entomologic nuisance and does not receive much attention and therefore remains an important public health problem for the poor. It is a problem neglected by those affected, the medical profession and the scientific community [3].

Household status determines the health conditions of the occupants and home hygiene is important in order to control pests and provide pleasant atmosphere to the household members [4]. Jigger menace has also led to school dropout and it is estimate that over 2 million people in Kenya need assistance in relation to jigger menace [5]. The risk of secondary infection is high. Tetanus is a common secondary infection that has reported associations with death [6].

Determinants of health include healthy human activities (practices, knowledge and attitude) as well as environmental determinants that create conditions which impact on the epidemiological pattern of these diseases and conditions. These determinants also increase susceptibility to environmental factors leading to more breeding sites for the vectors and increase the risk of jigger transmission [7].

If health workers are to deal effectively with ill community health, they need to understand their practices, ignorance, poverty and attitudes. They need to understand their behaviour and the surrounding environment in which people live [6]. Published information on knowledge, attitude and practices as well as jigger situation is scanty and fragmented in Kenya despite having a well focused National Health policies and reform agenda; consequently, there has not been a breakthrough in improving the situation of households entrapped in vicious cycle of poverty and ill health. This study therefore sought to elucidate the status of knowledge, attitude and practices on jigger infestation in a rural Kenyan setting [9].

## Methods

The study used descriptive cross sectional design where a rural area was purposely sampled to determine the knowledge, attitude and practices among household members, aged 18 to 60 years (the most affected age despite the level of maturity) on jigger infestation, prevention and control [10]. The area which is administratively divided in to eight villages (namely Rukoroi, Gakira, Kianjogu, Watuku, Gathanji, Rugaita, Gachagi and Githunguri) was purposively sampled due to jigger persistence. The households in the area formed the sampling frame where simple systematic sampling of the households was done and every 7th household was identified for the interview. A sample size of 277 was calculated but due to time constraints 271 (98.7%) respondents were interviewed in the study. A proportionate sample was then calculated using the total number of households per village.

**Data collection:** Data collection exercise was conducted for two weeks using a structured interview schedule. Housing, water storage facilities and environmental hygiene and sanitation status was also assessed using a checklist whereby the floor type, its cleanliness, water storage, and general compound maintenance were observed. The structured interview scheduled collected quantitative data on four thematic areas namely respondent's demographic characteristic, knowledge, attitude and practices through face to face interviews by the researcher and the research assistants. The knowledge on jigger infestation was measured by analyzing questions about; awareness of a jigger infested persons, ever suffered jigger infestation, who is the most affected in the community, what causes jigger, how jigger infestation is transmitted and jigger signs and symptoms, myths and misconception on jigger infestation. Attitude which is a subjective parameter was measured by seeking response toward individual and community perception of jigger infestation. Practices on jigger prevention and control as well as jigger infestation were measured using questions on; preferred methods of jigger removal, awareness of individual and communal jigger prevention activities and awareness of the communal activities planners, jigger infested persons seen at the time of the study and reasons for jigger persistence at this time and era.

**Study population:** The study targeted household members residing within the study area aged between 18 to 60. This comprised an active group that should be knowledgeable enough to prevent jigger infested among themselves and other household members.

**Inclusion and exclusion criteria:** Permanent resident members of the Sub-location aged 18 to 60 years were included in either the interview and non permanent residents and those below 18 and above 60 years were excluded from the study.

**Data analysis and presentation:** Data was analyzed using Statistical Package Social Science (SPSS) computer software Version 17.0. Categorical data was analyzed and presented in frequencies, proportions, percentages, measures of central tendency and dispersion. The association between jigger infestation and selected variables was done using Pearson's Chi-square test with significance levels set at alpha=0.05, and a p value at 95% confidence intervals analyzed.

**Ethical consideration:** The research proposal was approved by AMREF in conjunction with School of Public Health, Moi University. Further approval by the Ministry of Education, Science and Technology was granted. Eligible respondents who gave informed consent were interviewed. To enhance anonymity, no respondent unique identifier was used in the study. The researcher also sought authority to undertake the study in the area from all the relevant offices and community leaders.

## Results

### Demographic characteristics

Majority (67.9%) of respondent were female of whom 67.5% were married, 12.5% were single, 11.1% separated and 8.9% widowed. Almost all (92.6) were Christian. Analysis of level of education revealed 50.6% had primary level education. Only 8.1% had attained college education level and 14.4% had no formal education. The main source of income reported by about half (49.8%) of the respondents was farming. Salaried respondents and students were 6.3% and 4.4% respectively. The average monthly income was Ksh 2,810.00±1,290.75 with lowest income level of Ksh 800 per month (1 US dollar = Kshs. 82). The average age of the respondents was 40.35 years ±11.52 (95% CI 38.97 - 41.73) (Table 1).

**Table 1:** Socio-demographic characteristics of the respondents

Characteristic	Frequencies (n=271)	Percentage (%)
**Sex**		
Male	87	32.1
Female	184	67.9
**Marital status**		
Married	183	67.5
Single	34	12.5
Separated	30	11.1
Widowed	24	8.9
**Religion**		
Christian	251	92.6
Muslims	8	3.0
Others	12	4.4
**Level of education**		
None	39	14.4
Primary	137	50.6
Secondary	73	26.9
College	22	8.1
**Type of housing**		
Temporary	26	9.6
Semi permanent	148	54.6
Permanent	97	35.8
**Main source of income**		
Farming	135	49.8
Business	38	14.0
Casual work	69	25.5
Salaried	17	6.3
Students	12	4.4
**Age**		
20 to 29years	55	20.3
30 to 39 years	92	33.9
40 to 49 years	52	19.2
50 to 59 years	62	22.9
60 years and above	10	3.7
Mean age	40.4±11.52	
**Monthly income in Ksh:** (1 dollar = Kenyan shillings 78)		
Not earning (students)	12	4.4
Less than 1,000/=	91	33.5
1,000 to 1,999/=	54	19.9
2,000 to 2,999/=	37	13.5
3,000 to 3,999/=	31	11.4
4,000 to 4,999/=	25	9.2
Over 5,000/=	23	8.5
Average income	2,810±1,290.75	

### Knowledge

**Figure 1:**
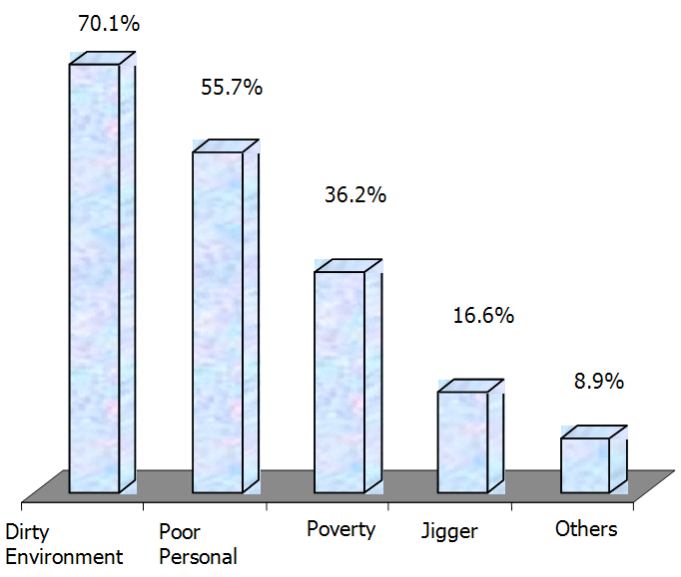
Causes of jigger infestation (Multiple responses - Percentage exceeds 100%)

The proportion of respondents who were aware of jigger infested persons was 88.9% while 92.7% had ever suffered jigger infestation. Children were reported to be the most (46.1%) affected persons. On jigger causes, 70.1% reported dirty environment, 55.7% poor personal hygiene, 36.2% poverty and 16.6% jigger flea (Figure 1). Over half (53.9%) reported jiggers are transmissible from person to person. The reported early jigger signs and symptoms were: frequent itching (94.8%), pain (14.1%), black pin head dot (10.7%), deformed lower limbs and coldness (3.3%). Almost half (48.7%) believed in one or more myths and misconception on jigger infestation. These included a curse (59.1%), poor people/households (43.2%), jiggers are a must in dry season (16.7%) and persons with specific blood group or specific families 3.8%.

### Attitude

Many (59.8%) reported jigger infested persons are lazy, 48.3% reported they are irresponsible, 26.2% reported they are poor while 12% reported they were either persons from specific families who must suffer infestation, people with certain blood group, elderly, illiterate or neglected children. The reasons for jigger persistence at this time and era were also sought where 79.3% reported poor hygiene and sanitation, 43.5% reported poverty, 9.6% reported varied reasons (that included illiteracy, drought and laziness), and 5.5% reported soil type while 2.6% reported jigger infestation is normal.

### Practices

The study revealed that the majority (88.2%) of the respondents had ever suffered jigger infestation in their life time. Over a quarter (23.6%) (64 households) reported between one to five household members suffering from jigger infestation. However, 18.8% (51) jigger infested persons were observed in the households (Table 2).

**Table 2:** Household members jigger experience and infestation

Characteristics	Frequencies (n=271)	Percentage (%)
Household Members Ever Suffer Jigger Infestation:		
Yes	239	88.2
Households reported with jiggers infested persons:		
Yes	64	23.6
Number of household members infested		
1 Member	40	14.8
2 Members	19	7.0
3 Members	4	1.5
5 Members	1	0.3
Number of households with jiggers infested persons seen during the study:		
Yes	51	18.8
Number of infested household members		
1Person	32	11.8
2 Persons	15	5.5
3 Persons	4	1.5

**Figure 2:**
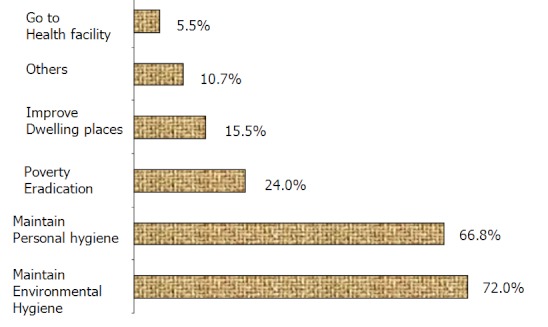
Jigger prevention and control measures (Multiple responses - Percentage exceeds 100%)

The most preferred jigger removal item used was needles/pin and thorns, reported by 90.8% and 38.7% respectively while 6.6% reported chemical washing. On individual jigger prevention and control activities, 72% reported maintenance of environmental hygiene, 66.8% reported maintenance of personal hygiene, 24% reported poverty eradication and 15.5% reported improvement of the dwelling place (Figure 2). Communal jigger prevention and control activities were not known by many (62%). Over half (59.8%) of those aware of communal prevention and control activities, 59.8% reported communal clean ups while 33.3% reported health education promotion. Most (48.5%) reported planners of communal prevention and control activities were the Health Extension Workers (CHEWs) followed by Community Health Workers (CHWs) 42.7%, then teachers/pupils and neighbours of infested persons 29.1% and 11.7% respectively.

Association test done on independent variables revealed that the respondent's village, (specifically Gathanje village) the type of floor and its status and the general maintenance of the compound were found to be significantly p < 0.05 (<0.001; 006; 0.004 and <0.001 respectively) associated with jigger infestation among household members (Table 3). The study revealed that, many (53.5%) of the floors was earthen with 45.8% of them poorly maintained. Most 59.6% of the households had a water reservoir with water. Less than half (42.4%) of the compounds were poorly maintained at the time of study (Table 4).

**Table 3:** Chi-Square test for variable association

Independent variable	Yes n (%)	No n (%)	Statistical test
**Village and current suffering members:**			
Gathanji	6 (20.0)	24	χ2 = 36.331; df 7;
		(80.0)	ρ < 0.05; (0.001)
**Type of floor and current jigger infestation:**			
Earthen	45	88	χ2 = 7.615; df 1;
	(33.8)	(66.2)	ρ < 0.05; (0.006)
**Floor maintenance and jigger infestation:**			
Not maintained	40	73	χ2 = 8.123; df 1;
	(35.4)	(64.6)	ρ < 0.05; (0.004)
**Compound maintenance and jigger infestation:**			
Poorly maintained	36.6%	63.4%	χ2 = 10.385; df 1
	(41)	(71)	ρ < 0.05; (0.001)

**Table 4:** Housing and environmental sanitation

Variable	Frequencies (n=271)	Percentage (%)
**Type of floor for the dwelling house:**		
Concrete	126	46.5
Earthen	145	53.5
**Status of the floor:**		
Well maintained	147	54.2
Dusty	117	43.2
Cracked	7	2.6
**Water storage facilities:**		
Present/seen	161	59.4
Absent/not seen	108	39.9
Piped supply seen	2	0.7
**General compound appearance:**		
Well maintained	156	57.6
Not maintained	115	42.4

## Discussion

The descriptive study design used allowed for a point in time assessment of the study variable where household member's knowledge, attitude and practices as well as jigger prevalence late were assessed. The rationale for the design was essentially due to the nature of the study and time allocated for the study.

The reported knowledge on jigger is relatively high. However, there has no related evidence for jigger prevention and control in the area. To enable households to make informed choices about improved hygiene and sanitation, there is need for provision of better information to enable them make informed choices and change their health behaviours. To provide such information, careful research needs to be done to identify feasible technical alternative and information gaps to suit the ability of both the Government and communities at large. The Government's goal is to achieve a major national wide impact on hygiene and sanitation related diseases by the year 2015 [3]. The knowledge on early signs and symptoms should act as a trigger mechanism for the household members to take appropriate action to prevent and control infestation. The first evidence of infestation by the sand flea is a tiny black dot on the skin at the point of penetration and then a small inflammatory papule with a central black dot forms within the next few weeks. Itching is the commonest symptoms [7]. Dusty surfaces, cracks and crevices in the walls and floors harbour insects and vermin therefore should be avoided. Home hygiene is important in order to control pests and provide pleasant atmosphere to the household members [10].

Though the respondents reported mixed attitude toward jigger infestation, the jigger problem is often brushed off as a thing of the past, or as a minor problem that can be relegated for more pressing issues. Public health experts warn that heavy jiggers infestations goes beyond mere discomfort and can leads to loss of toe/finger nails, amputation of the digits and could even cause death [17] which only exacerbate the problem further. It is therefore important to inculcate the right attitude in household members in order to develop a positive behaviour change for sustainable jigger prevention and control among household members. Electronic and print media has been revealing disturbing news about jigger's infestation in the study area to the effect that, some community members are infested to the point of being admitted in hospitals for treatment. This is disturbing owing to the fact that observing simple personal and household hygienic measures can prevent jiggers [16]. The high proportion of household members practicing jigger prevention and control activities as well as the CHEWs, CHWs, teachers and pupils’ efforts to undertake communal activities should translate to reduction and consequent eradication of jiggers in the area. Scientific knowledge on how to deal best with Tungiasis is scanty. Consequently, addressing comprehensive and sustainable solutions to these neglected health problems cannot be the sole responsibility of the health sector but it also requires community participation and multi-sectoral approaches to the health determinants [7]. In Central Province, a total of 1,350 persons suffered from jigger infestation in one Location in Murang'a District, of whom 700 were school going children. The study also revealed that 50% of the infested children do not attend classes [12]. Lives in some rural areas in Kenya revolves around jiggers as they are busy grappling with a problem Kenyans seem to ignore [11]. A campaign named “Operation Jigger Out” (OJO) indicated 103 serious cases of jigger infestation in the study area. A follow up report on jigger cases reported 285 jiggers infested household members. The report further revealed that pupils in six public schools assessed were also found to be infested with jiggers in the area [14].

Some of the reported reasons for jigger persistence in this study may contribute to continued jigger infestation in the area. Tungiasis is associated with poverty and occurs in resource poor countries in the Caribbean, South America and Africa [3]. People living in poverty throughout the world are heavily burdened from a series of communicable diseases and conditions particularly parasitic infestation [7]. Some of these factors concurred with findings [13] which assessed demographic and environmental characteristics of the population and showed that the occurrence of Tungiasis infestation was significantly (p < 0.05) related to poor housing conditions and lack of health education. The consequences of parasitic infestation control are part of a complex circle involving many socio-economic factors and should therefore not be measured without considering other related factors [10].

## Conclusion

In order to win the battle on jigger menace, it is important to recognize and use the existing knowledge and community structures for positive behaviour change in jigger prevention and control. It is important for the health workers to embrace the spirit of public and private partnership in order to implement sustainable hygiene practices to eradicate jigger in the area. It is also critical to inculcate the right attitude and empower household members to able to effectively prevent and control jigger infestation within the households for sustainable health and development. A continuous multi-sectoral approach and active community involvement and participation is also necessary to identify and address underlying causes to jigger infestation among the affected households in the area. Concerted efforts should be made to transform the community knowledge, attitude and practices to positive hygienic and sanitation behaviours/practices that will contribute to jigger eradication and consequently contributing to improved general health status and sustainable community development.
